# Developmental outcomes across foster care, adoption, and child welfare services: a mini review

**DOI:** 10.3389/fpsyt.2026.1691850

**Published:** 2026-01-29

**Authors:** Mireia Solerdelcoll, Gisela Sugranyes, Inmaculada Baeza

**Affiliations:** 1 Department of Child and Adolescent Psychiatry and Psychology, Hospital Clínic de Barcelona, Barcelona, Spain; 2 Institut d’Investigacions Biomèdiques August Pi Sunyer (IDIBAPS), Barcelona, Spain; 3 Department of Medicine, Institute of Neurosciences, Universitat de Barcelona, Barcelona, Spain; 4 Centro de Investigación Biomédica en Red de Salud Mental, CIBERSAM-ISCIII, Madrid, Spain

**Keywords:** adoption, child welfare services, developmental outcomes, education, foster care, mental health, placement stability, resilience

## Abstract

Children entering foster care (FC), adoption, or child welfare services (CWS) frequently experience early adversity, placing them at increased risk across multiple developmental domains. A structured narrative search (2020–October 2025) identified 61 eligible studies, including 22 high-quality longitudinal, administrative, and review-based ‘core’ studies. Findings were synthesised across five predefined domains to provide comparative evidence on placement stability, psychological and behavioural health, educational attainment, physical and developmental health, and social relationships. Findings indicate that FC is most consistently associated with instability and cumulative disadvantage, including high rates of mental health problems, disrupted education, unmet health needs, and relational difficulties. Adoption generally provides greater permanence and more favourable outcomes, particularly when it occurs early, though adoption at later ages or following institutional care is often linked to enduring emotional, behavioural, and neurodevelopmental difficulties. Children who remain with their families under CWS support show highly variable trajectories, with positive outcomes when interventions are timely and sustained, but persistent difficulties when family adversity continues and systemic support is lacking. Across pathways, placement stability—conceptualised as both an outcome domain and a protective process—consistently emerges, alongside secure relationships, trauma-informed approaches, and coordinated support, as a critical factor safeguarding children’s developmental outcomes. However, the evidence base remains limited, with few longitudinal, intervention-driven, and culturally inclusive studies. Addressing these gaps is essential to advance policy and practice reforms that promote more responsive and coordinated systems of care, enabling children to build resilience and achieve long-term wellbeing.

## 1 Introduction

Children entering foster care (FC), adoption, or child welfare services (CWS) frequently present with complex developmental and psychosocial needs, primarily attributable to adverse childhood experiences (ACEs) such as maltreatment, neglect, and family instability. These early adversities, often compounded by cumulative risk factors—including socioeconomic disadvantage and genetic vulnerability—are strongly associated with poorer outcomes in mental health, academic achievement, physical health, and social functioning (1). Although all three care systems aim to safeguard children’s wellbeing, the developmental trajectories they offer diverge markedly (2). Foster care, conceived as a temporary protective intervention, often entails placement disruptions and multiple caregiver changes. Adoption provides legal and emotional permanence, yet adjustment difficulties and identity-related challenges can persist, particularly among children adopted at older ages or following institutional care (e.g., orphanages or large group residential facilities). CWS interventions aim to preserve children within their biological families, promoting relational continuity. However, when family instability persists or systemic supports are under-resourced, children may remain exposed to ongoing risks (3). Children’s developmental trajectories are shaped not only by the care pathway but also by cumulative early adversity, the stability and quality of caregiving, living arrangements, and the adequacy of systemic support.

While previous reviews have typically focused on outcomes within specific groups—such as children in FC (4) or international adoptees (5)—few studies have compared these pathways. A comparative approach is essential to identify both shared challenges and unique risks, and to inform evidence-based interventions and policy reforms.

This review synthesises recent evidence across five key domains: placement stability, psychological and behavioural health, educational attainment, physical and developmental health^1^, and social relationships and support networks, to provide an integrated perspective on outcomes among children involved in FC, adoption, and CWS.

## 2 Methodology

This mini review employed a structured narrative synthesis, guided by PRISMA principles adapted for concise evidence syntheses. The aim was to integrate peer-reviewed research published between January 2020 and October 2025 on developmental outcomes among children involved in FC, adoption, or CWS across five predefined domains. The review period was selected to capture the most recent evidence, reflecting evolving child welfare policies, post-COVID service adaptations, and the emergence of new longitudinal research.

### 2.1 Search strategy

Comprehensive searches were conducted in PubMed/MEDLINE, PsycINFO, Web of Science Core, and Scopus, complemented by manual searches in Google Scholar and the reference lists of included articles. Search strings combined care-context and outcome-related keywords, for example: (“foster care” OR “out-of-home care” OR adoption OR “child welfare”) AND (placement OR stability OR “mental health” OR behaviour* OR education* OR development* OR “social relationships”). Searches were restricted to studies published in English or Spanish within the specified period.

### 2.2 Eligibility criteria

Studies were included if they were empirical investigations (cross-sectional, longitudinal, administrative, or register-based) or systematic reviews/meta-analyses that (a) examined children or adolescents (< 21 years) with experience of FC, adoption, or CWS/family-preservation services, and (b) reported outcomes within at least one of the five domains. Both comparative and high-quality single-group studies were eligible. Exclusion criteria comprised theoretical papers, case studies with very small samples, purely qualitative studies lacking outcome data, and intervention trials without baseline developmental results.

### 2.3 Screening, data extraction, and synthesis

Following deduplication, 782 records were screened by title and abstract, and 92 full-text articles were assessed for eligibility. A total of 61 studies met the inclusion criteria and were integrated into the narrative synthesis. Extracted data included study design, geographical context, sample characteristics, care pathway, and principal findings. Given the methodological heterogeneity across studies, findings were synthesised narratively and organised within the five predefined domains. Greater interpretative weight was accorded to systematic reviews, meta-analyses, and longitudinal population-based studies. Systematic reviews were appraised using AMSTAR-2 (A Measurement Tool to Assess Systematic Reviews) (6) and empirical studies using the Joanna Briggs Institute (JBI) critical appraisal tools. Quality appraisal informed interpretation but did not determine inclusion.

Twenty-two studies were designated as ‘core’ evidence, representing the most methodologically robust and conceptually comprehensive sources. These comprised longitudinal population-based cohorts, high-quality systematic reviews and meta-analyses, and large administrative or registry-based studies published predominantly between 2020 and 2025. One foundational longitudinal cohort from 2017 was retained because it provides unique developmental follow-up data essential for interpreting later research. All studies were screened using the same eligibility criteria, and the ‘core’ designation was applied post-inclusion to identify the strongest evidence. Core studies were not analysed separately but received greater interpretative weight during synthesis, consistent with the aims of a mini review. **Supplementary Table S1** summarises these core studies to enhance transparency.

## 3 Results

### 3.1 Placement stability

Placement stability refers to the continuity of a child’s living arrangements in care. Stability is critical for secure attachment and healthy development, whereas frequent moves disrupt relationships, schooling, and access to services (7). Findings from the reviewed studies highlight that consistent caregiving, which provides permanence and security, is associated with more favourable long-term outcomes (8, 9).

Placement instability is common in FC, with longitudinal and administrative studies from several countries showing frequent multiple moves, particularly among older children and those with behavioural difficulties (10–13). Each additional move is associated with weaker attachment formation, disrupted schooling, and heightened risk of mental health problems (4, 7). Adoption generally provides greater permanence, particularly when placement occurs early in life (14). Nonetheless, children adopted at older ages or internationally may experience persistent adjustment difficulties due to prior trauma and loss (5, 15). Children who remain with their families receiving CWS support may benefit from stability when preventive, in-home interventions are timely and sustained. However, without adequate support, ongoing family instability may lead to breakdown and eventual foster placement, undermining developmental continuity (16, 17).

Overall, adoption provides the highest stability, followed by well-supported CWS family-preservation programmes, whereas FC remains most often associated with instability and its adverse consequences (18, 19). Across care systems, recent longitudinal and meta-analytic evidence converges on the central role of placement continuity, carer preparation, and ongoing professional support as key determinants of permanence and developmental wellbeing (8, 9, 13).

### 3.2 Psychological and behavioural health

This domain encompasses emotional wellbeing, mental health, and behavioural adjustment. Evidence from the reviewed studies indicates that children involved in FC, adoption, or CWS are at significantly higher risk of psychological and behavioural difficulties than their peers (20, 21). Early trauma and unstable caregiving environments increase the likelihood of psychological difficulties, making the promotion of mental health a critical developmental priority across all care pathways (1, 22). It is also important to consider that pre-existing mental health difficulties may contribute to child welfare involvement, particularly FC entry, and may therefore act as a potential confounder in interpreting these associations.

Youth in FC exhibit the highest prevalence of psychological and behavioural problems, with 50–60% meeting criteria for at least one psychiatric disorder (21). These difficulties arise from cumulative adversities, including abuse, neglect, parental mental illness, and frequent placement changes (23). Placement instability is strongly associated with poorer mental health outcomes, predicting both externalising and internalising symptoms, whereas stable placements are linked to improved emotional and behavioural regulation (4). Psychotropic medication use—often involving polypharmacy with limited oversight—remains disproportionately high in this group (24, 25), A retrospective cohort study from Catalonia (Spain) reported elevated rates of conduct disorder, substance misuse, and psychiatric comorbidity among hospitalised foster youth compared with community peers, underscoring the complexity of their needs (26). Adopted children generally exhibit more favourable psychological outcomes, particularly when adoption occurs early within stable, nurturing families. Early adoption can mitigate many risks, with children adopted before age two often achieving outcomes comparable to non-adopted peers (27). In contrast, late adoption—particularly from institutional care—is associated with attachment insecurity, identity-related stress, and persistent emotional and behavioural difficulties, often intensifying during adolescence (15, 28, 29). Longitudinal findings from the English and Romanian Adoptee (ERA) study indicate that children exposed to over six months of severe institutional deprivation exhibit a distinctive and enduring neurodevelopmental profile, including quasi-autism, inattention/hyperactivity, and disinhibited social engagement—traits that persist into adulthood despite adoption into supportive families (27, 30). Children receiving CWS support show variable mental health trajectories. Longitudinal analyses reveal that secure caregiver–child relationships buffer behavioural risk among adolescents under CWS supervision (17). When interventions such as parenting support, family therapy, and parental mental health treatment are provided in a consistent and well-coordinated manner, outcomes for children and adolescents under CWS supervision can approach those of non-involved peers. However, fragmented or insufficient services often leave major needs unmet, allowing family dysfunction to persist (20, 22, 31). Prolonged exposure to maltreatment or domestic instability without adequate support can lead to cumulative psychological harm (32).

Across all care pathways, children and adolescents face elevated mental health risks compared with peers without child welfare involvement, with foster youth consistently the most affected. The limited availability of longitudinal and comparative research on mental health trajectories across care pathways constrains the development of sustained, developmentally targeted services. An integrated, cross-sector approach to mental health care is essential to address the complex needs of this population.

### 3.3 Educational attainment

Educational attainment reflects not only academic achievement but also the role of schools as protective environments that provide socioemotional support, daily structure, access to basic needs, and early detection of developmental or behavioural difficulties (33, 34). The reviewed evidence demonstrates that educational attainment remains one of the most persistent inequalities across care pathways. Poor educational outcomes perpetuate intergenerational disadvantage, whereas educational attainment is associated with better adult employment, income stability, and overall wellbeing (35).

Youth in FC consistently demonstrate lower educational attainment than their peers, with elevated rates of absenteeism, grade repetition, and early school leaving (36). Placement instability, frequent school changes, and inconsistent educational advocacy further compound these disadvantages (33, 34). Longitudinal and administrative data indicate that only around half of youth in FC complete secondary education on time, compared with over 80% of non-involved peers (37, 38). Access to and completion of tertiary education remains disproportionately low (39). These gaps often persist into adulthood, contributing to socioeconomic marginalisation (39). Adopted children generally achieve more favourable educational outcomes, especially when adoption occurs early and is supported by stable families and consistent schooling. In contrast, adoption following prolonged institutional care is often associated with persistent academic underachievement and learning difficulties, including language delays, executive dysfunction, and neurodevelopmental impairments, commonly associated with early deprivation or prenatal exposure (5, 14, 15). Evidence from a national English cohort shows that early entry into FC predicts more stable educational trajectories and better academic outcomes (40). Children receiving CWS support show highly heterogeneous academic outcomes. When interventions—such as targeted tutoring, mentoring, and parental engagement—are timely, sustained, and well-coordinated, educational outcomes can approach those of non-involved peers (41). However, persistent family adversity (e.g., parental substance misuse, domestic violence, or chronic poverty), combined with low parental engagement and inadequate intervention, increases the risk of truancy, academic delay, and low aspirations (42).

In summary, educational continuity and targeted support are central to reducing disparities. Across all reviewed studies, children and adolescents involved in child protection systems consistently showed lower educational attainment than their non-involved peers, with foster youth experiencing the greatest disadvantage. Early adoption, sustained family-preservation support, and policies that protect school stability were associated with improved educational outcomes (42).

### 3.4 Physical and developmental health

This domain encompasses physical health, growth, and neurodevelopment. Across the reviewed studies, children involved in FC, adoption, and CWS consistently showed higher rates of physical health problems and developmental difficulties than their peers. These disparities are rooted in early adversity—including prenatal substance exposure, neglect, malnutrition, and chronic stress—and are compounded by inconsistent access to healthcare and fragmented service delivery (43). Genetic and neurobiological factors, including specific learning disabilities and language disorders, may further contribute to developmental difficulties in some children (44, 45).

Youth in FC present elevated rates of chronic health conditions, developmental delays, and unmet medical needs compared with peers without child welfare involvement (44, 45). Youth in FC youth experience the greatest burden, with up to 80% requiring treatment for conditions such as asthma, malnutrition, or dental disease (21, 46). Placement instability further disrupts continuity of care, while incomplete medical histories and delayed referrals hinder timely assessment and intervention (46, 47). Adolescents in FC are also more likely to engage in health-risky behaviours, including early sexual activity and substance use, with associated long-term health consequences (26, 47). Meta-analytic evidence indicates that adopted children demonstrate significant post-placement catch-up in growth and cognitive development, although complete recovery from early deprivation is uncommon (48). Children adopted after prolonged institutional care often display persistent developmental difficulties, including growth stunting, immune dysregulation, and neurodevelopmental conditions such as foetal alcohol spectrum disorder (49, 50). Children under CWS supervision show highly variable health outcomes, shaped by healthcare access and family stability. Persistent socioeconomic adversity, systemic under-resourcing, and competing priorities often delay diagnosis and treatment, increasing the risk of chronic illness and developmental delay (51). Analyses of linked administrative data reveal persistent health-service utilisation across successive family generations engaged with CWS, underscoring intergenerational vulnerability and the imperative for sustained cross-sector coordination (31).

Overall, youth in FC experience a disproportionate burden of physical and developmental health difficulties (21, 43). Early and stable adoption can promote partial recovery; however, the developmental consequences of early deprivation and perinatal complications often persist (14). Comprehensive health and developmental screening, timely multidisciplinary intervention, and sustained collaboration between welfare and healthcare systems are essential to improving outcomes across all care pathways (31, 51).

### 3.5 Social relationships and support networks

This domain encompasses the quality and stability of children’s relationships with caregivers, siblings, and peers, as well as access to broader social support and a sense of belonging. Across the reviewed studies, secure and nurturing relationships consistently emerged as key protective factors, promoting resilience, emotional regulation, and identity development among children involved in child welfare systems. In contrast, disrupted or conflictual relationships can undermine emotional wellbeing, behavioural adjustment, and social integration. (52, 53).

Foster youth often experience significant relational disruption through caregiver changes, sibling separation, and limited family contact. These disruptions can weaken attachment security, hinder trust formation, and contribute to social withdrawal, and feelings of marginalisation and loneliness. Adolescence represents a particularly sensitive developmental period during which stigma, school mobility, and behavioural difficulties may further erode protective social networks, heightening vulnerability to isolation, identity confusion, and psychosocial maladjustment (4, 7, 54). Longitudinal evidence indicates that only about half of care-experienced young people maintain an enduring adult relationship after leaving care, and those who do demonstrate greater resilience and a reduced risk of homelessness (55). Adopted children generally benefit from stable, long-term caregiving relationships, especially when adoption occurs early in supportive families. However, those adopted later or following institutional care may struggle with attachment insecurity, identity-related challenges, and unresolved loss, which can resurface during adolescence (5, 14). Evidence suggests that open adoption models and culturally responsive post-adoption support can enhance relational wellbeing and identity integration (56–58). Children and families under CWS supervision often retain family and community connections, which can promote cultural identity, belonging, and social stability. However, when family adversity remains unresolved, these relationships may instead become sources of conflict or emotional strain. Limited access to extracurricular activities, unsafe neighbourhoods, parental mental illness, and exposure to risky behaviours can further constrain social opportunities (59). Inconsistent professional support and limited access to structured peer or community programmes can heighten isolation and hinder the development of supportive relationships (17).

Across all care pathways, secure and enduring relationships—both within and outside the family—consistently emerge as key protective factors of resilience. Although the evidence remains limited, interventions such as peer mentoring, sibling contact, attachment-focused therapies, and community engagement programmes show promise for improving social outcomes (60–62).

## 4 Discussion

This review synthesises evidence on developmental outcomes among children involved in FC, adoption, and CWS, identifying shared challenges and distinct developmental trajectories. Across domains, early adversity, placement instability, and fragmented service provision emerge as key risk factors, whereas overall stability, timely intervention, and effective cross-sector collaboration consistently act as protective influences (63–65). Despite cross-national and systemic differences, the reviewed studies converge on several cross-cutting mechanisms that consistently shape children’s developmental trajectories.

Placement and relational stability consistently emerge as central protective processes influencing wellbeing across mental health, education, and social development. Continuity of caregiving, early permanency, and supportive family environments are associated with more favourable outcomes, whereas placement instability and disruption, and fragmented care networks increase developmental risk (7, 11, 12, 26, 42). Early adoption and well-coordinated family-preservation support under CWS promote better outcomes, although the developmental consequences of early deprivation, perinatal complications, and chronic adversity frequently persist despite later stability (31, 65). Longitudinal evidence confirms that placement stability predicts better socio-emotional, cognitive, and health outcomes across child welfare trajectories (8, 9, 13).

Fragmentation between welfare, health, and educational services remains a major obstacle to continuity of care, delaying the identification of emerging needs and reducing the effectiveness of interventions (37, 54, 66). Even where trauma-informed or resilience-based approaches have been implemented, inconsistent professional training, high staff turnover, and limited cross-sector coordination undermine their long-term impact.

Across pathways, foster youth consistently represent the most vulnerable group, displaying the highest rates of mental health disorders, educational underachievement, and physical health difficulties (26, 42, 59). Adopted children generally demonstrate partial recovery—particularly when placed early in stable, well-supported families—yet the enduring effects of early deprivation and perinatal risk often limit full remediation (25, 49, 67). Children and families receiving CWS support display highly variable trajectories, with outcomes largely determined by the intensity, duration, and quality of community-based interventions (17, 31).

Overall, the evidence underscores the critical importance of integrated, trauma-informed, and developmentally responsive systems of care. Strengthened collaboration among welfare, education, and health sectors is essential to promote stability, resilience, and recovery among children and families affected by early adversity (39, 42, 57). Yet, the persistence of service fragmentation and the limited longitudinal, intervention-focused research continue to constrain progress. Recent co-produced and longitudinal studies demonstrate that coordinated, multi-agency approaches and early permanency planning yield the most durable improvements in mental-health, educational, and social outcomes (38, 66). Addressing these gaps through coordinated, evidence-driven reforms is fundamental to ensuring that all children—irrespective of care pathway—can achieve stable relationships and equitable developmental outcomes. The following section outlines key evidence gaps and policy priorities arising from this synthesis.

## 5 Cross-cutting gaps and policy recommendations

Comparisons across care pathways are limited by heterogeneous study designs, inconsistent outcome measures, and reliance on retrospective or cross-sectional data. Longitudinal, intervention-focused, and cross-national research remains scarce—particularly in non-Anglophone and Southern European contexts—leading to the underrepresentation of diverse cultural settings and limiting the generalisability of findings (18, 68). Few studies adequately control for pre-placement adversity, including pre-existing mental health difficulties, prenatal exposures, or early developmental delays, which may act as confounders when interpreting pathway differences. In addition, limited attention has been given to protective factors, resilience processes, and the lived perspectives of children and families, and few studies examine the long-term impact of targeted interventions (69).

Bridging these evidence gaps requires translating comparative insights into actionable policy and practice reforms. Reforms should prioritise protective factors shared across care systems, focusing on stability, continuity, and a sense of belonging, while embedding integrated and culturally responsive services that buffer risk and promote resilience.

To address these gaps, key policy and practice priorities include:

- Promoting placement stability: Minimise placement disruptions in FC and prioritise family-based placements when removal is necessary; ensure early permanency in adoption; and strengthen in-home support for families under CWS to prevent unnecessary removals.
- Integrating services: Foster coordination between child welfare, education, and health sectors to deliver timely, holistic, and continuous care.
- Ensuring health screening and follow-up: Mandate comprehensive health and developmental assessments at entry into care, followed by ongoing monitoring and specialist referral.
- Safeguarding school continuity: Ensure educational stability during placement transitions and provide individualised educational planning.
- Supporting relationships: Preserve sibling bonds, sustain trusted adult connections, and expand mentoring, family therapies, and community-based programmes.
- Enhancing cultural responsiveness: Embed practices that preserve cultural identity, belonging, and community ties, tailored to the distinct needs and sociocultural contexts of each care system.
- Strengthening longitudinal research: Prioritise prospective, cross-pathway studies that follow children into adulthood to evaluate the long-term impact of interventions.
- Building cross-sector infrastructure: Develop joint frameworks, shared outcome measures, and interdisciplinary training to embed collaboration across welfare, health, and education systems.

Together, these priorities emphasise stability, family-based care, and integrated services as the foundation for systemic reform.

## 6 Conclusion

This review underscores the importance of a comparative framework for understanding developmental trajectories across child welfare pathways. Outcomes for children in FC, adoption, and CWS-supervised family preservation are shaped not only by the pathway itself but also by placement histories, caregiving quality, and cumulative exposure to adversity (7).

Across systems, stability, enduring relationships, timely identification of needs, and effective cross-sector collaboration consistently emerge as protective factors, whereas instability, early deprivation, and fragmented care contribute to development risks and long-term disadvantage (70).

Future research must move beyond cross-sectional comparisons toward longitudinal, intervention-oriented, and culturally inclusive designs that capture resilience processes and the lived experiences of children and families. At the policy level, embedding integrated, culturally responsive, and child-centred approaches across welfare, education, and health systems is essential to reducing disparities and supporting long-term wellbeing (18, 31).

Ultimately, comparative evidence should inform reforms that ensure all children grow up in stable, nurturing environments that foster security, resilience, and healthy development across the lifespan.

## Footnotes

1 The distinction between physical and developmental health is retained because the reviewed studies operationalise these constructs separately: physical health refers to medical conditions and healthcare access, whereas developmental health captures growth and neurodevelopmental outcomes.

## References

[1] Turney K Wildeman C . Adverse childhood experiences among children placed in and adopted from foster care: Evidence from a nationally representative survey. Child Abuse Negl. (2017) 64:117–29. doi: 10.1016/j.chiabu.2016.12.009, PMID: 28086178

[2] van IJzendoorn MH Bakermans-Kranenburg MJ Duschinsky R Fox NA Goldman PS Gunnar MR . Institutionalisation and deinstitutionalisation of children 1: a systematic and integrative review of evidence regarding effects on development. Lancet Psychiatry. (2020) 7:703–20. doi: 10.1016/S2215-0366(19)30399-2, PMID: 32589867

[3] LaBrenz CA Childress S Robinson ED Sieger ML Ontiberos J . Reasonable efforts to preserve families? An examination of service utilization and child removal. Child Abuse Negl. (2022) 128:105631. doi: 10.1016/j.chiabu.2022.105631, PMID: 35417852 PMC9728499

[4] Maguire D May K McCormack D Fosker T . A systematic review of the impact of placement instability on emotional and behavioural outcomes among children in foster care. J Child Adolesc Trauma. (2024) 17:641–55. doi: 10.1007/s40653-023-00606-1, PMID: 38938940 PMC11199447

[5] Juffer F Palacios J Le Mare L Sonuga-Barke EJS Tieman W Bakermans-Kranenburg MJ . Development of adopted children with histories of early adversity. Monogr Soc Res Child Dev. (2011) 76:31–61. doi: 10.1111/j.1540-5834.2011.00627.x

[6] Shea BJ Reeves BC Wells G Thuku M Hamel C Moran J . AMSTAR 2: a critical appraisal tool for systematic reviews that include randomised or non-randomised studies of healthcare interventions, or both. BMJ. (2017) 358:j4008. doi: 10.1136/bmj.j4008, PMID: 28935701 PMC5833365

[7] Rubin DM O’Reilly ALR Luan X Localio AR . The impact of placement stability on behavioral well-being for children in foster care. Pediatrics. (2007) 119:336–44. doi: 10.1542/peds.2006-1995, PMID: 17272624 PMC2693406

[8] Vanderwill LA Salazar AM Jenkins G Larwelle J McMahon AK Day A . Systematic literature review of foster and adoptive caregiver factors for increasing placement stability and permanency. J Public Child Welf. (2021) 15:487–527. doi: 10.1080/15548732.2020.1760176

[9] Asif N Breen C Wells R . Influence of placement stability on developmental outcomes of children and young people in out-of-home care: Findings from the Pathways of Care Longitudinal Study. Child Abuse Negl. (2024) 149:106145. doi: 10.1016/j.chiabu.2023.106145, PMID: 37003854

[10] Connell CM Vanderploeg JJ Flaspohler P Katz KH Saunders L Tebes JK . Changes in placement among children in foster care: A longitudinal study of child and case influences. Soc service Rev (Chicago). (2006) 80:398–418. doi: 10.1086/505554, PMID: 25342863 PMC4204626

[11] Mc Grath-Lone L Harron K Dearden L Gilbert R . Exploring placement stability for children in out-of-home care in England: a sequence analysis of longitudinal administrative data. Child Abuse Negl. (2020) 109:104689–13. doi: 10.1016/j.chiabu.2020.104689, PMID: 32891970 PMC7613165

[12] Stenason L Romano E . Number of placement changes among young people in care: Youth and caregiver associations. Child Youth Serv Rev. (2023) 144:106737. doi: 10.1016/j.childyouth.2022.106737

[13] Eltink EMA Waaijenberg A Broers M van Anrooij M van Rooij FB Stams GJJM . The prevalence of placement breakdown in foster care: A meta-analysis. Child Youth Serv Rev. (2025) 171:108203. doi: 10.1016/j.childyouth.2025.108203

[14] van IJzendoorn MH Palacios J Sonuga-Barke EJS Gunnar MR Vorria P McCall RB . Children in institutional care: delayed development and resilience. Monogr Soc Res Child Dev. (2011) 76:8–30. doi: 10.1111/j.1540-5834.2011.00626.x, PMID: 25125707 PMC4130248

[15] Palacios J Adroher S Brodzinsky DM Grotevant HD Johnson DE Juffer F . Adoption in the service of child protection: an international interdisciplinary perspective. Psychology Public policy Law. (2019) 25:57–72. doi: 10.1037/law0000192

[16] Font SA Sattler KMP Gershoff E . When home is still unsafe: from family reunification to foster care reentry. J marriage Family. (2018) 80:1333–43. doi: 10.1111/jomf.12499, PMID: 30479404 PMC6251317

[17] Yoon S Sattler K Knox J Xin Y . Longitudinal examination of resilience among child welfare-involved adolescents: The roles of caregiver–child relationships and deviant peer affiliation. Dev Psychopathol. (2023) 35:1069–78. doi: 10.1017/s0954579421000924, PMID: 34766899 PMC9345746

[18] del Valle JF Bravo A . Current trends, figures and challenges in out of home child care: An international comparative analysis. Interv Psicosoc. (2013) 22:251–7. doi: 10.5093/in2013a28

[19] Lindner AR Hanlon R . Outcomes of youth with foster care experiences based on permanency outcome – Adoption, aging out, long-term foster care, and reunification: A systematic review. Child Youth Serv Rev. (2024) 156:107366. doi: 10.1016/j.childyouth.2023.107366

[20] Bronsard G Alessandrini M Fond G Loundou A Auquier P Tordjman S . The prevalence of mental disorders among children and adolescents in the child welfare system: A systematic review and meta-analysis. Med (Baltimore). (2016) 95:e2622–2. doi: 10.1097/MD.0000000000002622, PMID: 26886603 PMC4998603

[21] Szilagyi MA Rosen DS Rubin D Zlotnik S Harmon D Jaudes P . Health care issues for children and adolescents in foster care and kinship care. Pediatr (Evanston). (2015) 136:e1142–66. doi: 10.1542/peds.2015-2656, PMID: 26416934

[22] Burns BJ Phillips SD Wagner HR Barth RP Kolko DJ Campell Y . Mental health need and access to mental health services by youths involved with child welfare: A national survey. J Am Acad Child Adolesc Psychiatry. (2004) 43:960–70. doi: 10.1097/01.chi.0000127590.95585.65, PMID: 15266190

[23] Segura A Pereda N Guilera G Abad J . Poly-victimization and psychopathology among Spanish adolescents in residential care. Child Abuse Negl. (2016) 55:40–51. doi: 10.1016/j.chiabu.2016.03.009, PMID: 27082753

[24] Zito JM Safer DJ Sai D Gardner JF Thomas D Coombes P . Psychotropic medication patterns among youth in foster care. Pediatrics. (2008) 121:e157–63. doi: 10.1542/peds.2007-0212, PMID: 18166534

[25] McLeigh JD Malthaner LQ Tovar MC Khan M . Mental health disorders and psychotropic medication: prevalence and related characteristics among individuals in foster care. J Child Adolesc Trauma. (2023) 16:745–57. doi: 10.1007/s40653-023-00547-9, PMID: 37593050 PMC10427591

[26] Solerdelcoll M Ilzarbe D Fortea A Morer A Lazaro L Sugranyes G . Psychopathology and mental health service use among youth in foster care admitted to a psychiatric inpatient unit: a 4-year retrospective controlled study. Eur Child Adolesc Psychiatry. (2024) 33:39–50. doi: 10.1007/s00787-022-02104-5, PMID: 36542199 PMC9768764

[27] Sonuga-Barke EJS Kennedy M Kumsta R Knights N Golm D Rutter M . Child-to-adult neurodevelopmental and mental health trajectories after early life deprivation: the young adult follow-up of the longitudinal English and Romanian Adoptees study. Lancet. (2017) 389:1539–48. doi: 10.1016/S0140-6736(17)30045-4, PMID: 28237264

[28] Juffer F van IJzendoorn MH . Behavior problems and mental health referrals of international adoptees: A meta-analysis. JAMA: J Am Med Assoc. (2005) 293:2501–15. doi: 10.1001/jama.293.20.2501, PMID: 15914751

[29] Brodzinsky D Gunnar M Palacios J . Adoption and trauma: Risks, recovery, and the lived experience of adoption. Child Abuse Negl. (2022) 130:105309. doi: 10.1016/j.chiabu.2021.105309, PMID: 34544593 PMC8926933

[30] Rodriguez-Perez M Kennedy M Barker ED Kreppner J Solerdelcoll M Sonuga-Barke EJS . The adult outcome of childhood quasi-autism arising following extreme institutional deprivation. J Child Psychol Psychiatry. (2023) 64:1292–1302. doi: 10.1111/jcpp.13767, PMID: 36782398 PMC10476691

[31] McKenzie EF Thompson CM Ogilvie JM Tzoumakis S Hurren E . Examining mental health service use across intergenerational patterns of child protection system contact: A case for cross-sector supports. Child Abuse Negl. (2025) 163:107426. doi: 10.1016/j.chiabu.2025.107426, PMID: 40112653

[32] Norman RE Byambaa M De R Butchart A Scott J Vos T . The long-term health consequences of child physical abuse, emotional abuse, and neglect: A systematic review and meta-analysis. PloS Med. (2012) 9:e1001349. doi: 10.1371/journal.pmed.1001349, PMID: 23209385 PMC3507962

[33] Solerdelcoll M . A wake-up call: recognizing and reimaging responses to children’s mental health and protection needs as an integral part of the COVID-19 pandemic. Front Psychiatry. (2022) 13:841515. doi: 10.3389/fpsyt.2022.841515, PMID: 35222129 PMC8866847

[34] O’Higgins A Sebba J Gardner F . What are the factors associated with educational achievement for children in kinship or foster care: A systematic review. Child Youth Serv Rev. (2017) 79:198–220. doi: 10.1016/j.childyouth.2017.06.004

[35] Pears KC Kim HK Buchanan R Fisher PA . Adverse consequences of school mobility for children in foster care: A prospective longitudinal study. Child Dev. (2015) 86:1210–26. doi: 10.1111/cdev.12374, PMID: 25906815 PMC4618793

[36] McNamara P Montserrat C Wise S . Education in out-of-home care : international perspectives on policy, practice and research. 1st ed. Wise S McNamara P Montserrat C , editors. Cham: Springer (2019). doi: 10.1007/978-3-030-26372-0

[37] Sutcliffe AG Gardiner J Melhuish E . Educational progress of looked-after children in England: A study using group trajectory analysis. Pediatr (Evanston). (2017) 140:e20170503. doi: 10.1542/peds.2017-0503, PMID: 28778858

[38] Okpych NJ Whitman K Lee J Neria-Piña L Jackson LA Day M . Secondary and postsecondary education outcomes of students with experience in foster care: systematic review of the literature from 2000–2023. AERA Open. (2025) 11:23328584251331456. doi: 10.1177/23328584251331454

[39] Vinnerljung B Hjern A . Cognitive, educational and self-support outcomes of long-term foster care versus adoption. A Swedish national cohort study. Child Youth Serv Rev. (2011) 33:1902–10. doi: 10.1016/j.childyouth.2011.05.016

[40] Melkman EP . Educational trajectories of children in care across the early education and primary school years: A national cohort study in England. Am J orthopsychiatry. (2020) 90:720–32. doi: 10.1037/ort0000505, PMID: 32673025

[41] Sanders JE Fallon B . Child welfare involvement and academic difficulties: Characteristics of children, families, and households involved with child welfare and experiencing academic difficulties. Child Youth Serv Rev. (2018) 86:98–109. doi: 10.1016/j.childyouth.2018.01.024

[42] Forsman H Vinnerljung B . Interventions aiming to improve school achievements of children in out-of-home care: A scoping review. Child Youth Serv Rev. (2012) 34:1084–91. doi: 10.1016/j.childyouth.2012.01.037

[43] Turney K Wildeman C . Mental and physical health of children in foster care. Pediatrics. (2016) 138:e20161118. doi: 10.1542/peds.2016-1118, PMID: 27940775

[44] Stein REK Hurlburt MS Heneghan AM Zhang J Rolls-Reutz J Silver EJ . Chronic conditions among children investigated by child welfare: A national sample. Pediatr (Evanston). (2013) 131:455–62. doi: 10.1542/peds.2012-1774, PMID: 23420907 PMC4074665

[45] Kaferly J Orsi-Hunt R Hosokawa P Sevick C Creel LM Mathieu S . Health differs by foster care eligibility: A nine-year retrospective observational study among medicaid-enrolled children. Acad Pediatr. (2024) 24:1092–100. doi: 10.1016/j.acap.2023.12.006, PMID: 38142889

[46] Leslie LK Hurlburt MS Landsverk J Rolls JA Wood PA Kelleher KJ . Comprehensive assessments for children entering foster care: A national perspective. Pediatr (Evanston). (2003) 112:134–42. doi: 10.1542/peds.112.1.134, PMID: 12837879 PMC1519418

[47] Carpenter SC Clyman RB Davidson AJ Steiner JF . The association of foster care or kinship care with adolescent sexual behavior and first pregnancy. Pediatr (Evanston). (2001) 108:753–3. doi: 10.1542/peds.108.3.e46, PMID: 11533364

[48] Leroy JL Angel MD Frongillo EA . Adoption or placement in foster care and catch-up in linear growth and development: A meta-analysis of individual participant data. Adv Nutr. (2025) 16:100395. doi: 10.1016/j.advnut.2025.100395, PMID: 39993655 PMC11957773

[49] Gunnar MR Van Dulmen MHM . Behavior problems in postinstitutionalized internationally adopted children. Dev Psychopathol. (2007) 19:129–48. doi: 10.1017/S0954579407070071, PMID: 17241487

[50] Carrera P Miller LC Palacios J Román M . Psychosocial, neurocognitive, and physical development in Eastern European adopted adolescents with and without fetal alcohol spectrum disorder. Alcohol Clin Exp Res. (2025) 49:1248–62. doi: 10.1111/acer.70068, PMID: 40288910 PMC12174492

[51] Leslie LK Gordon JN Meneken L Premji K Michelmore KL Ganger W . The physical, developmental, and mental health needs of young children in child welfare by initial placement type. J Dev Behav Pediatr. (2005) 26:177–85. doi: 10.1097/00004703-200506000-00003, PMID: 15956866 PMC1550710

[52] Luthar SS Eisenberg N . Resilient adaptation among at-risk children: harnessing science toward maximizing salutary environments. Child Dev. (2017) 88:337–49. doi: 10.1111/cdev.12737, PMID: 28144962

[53] Ungar M . Resilience after maltreatment: The importance of social services as facilitators of positive adaptation. Child Abuse Negl. (2013) 37:110–5. doi: 10.1016/j.chiabu.2012.08.004, PMID: 23260114

[54] Wojciak AS McWey LM Waid J . Sibling relationships of youth in foster care: A predictor of resilience. Child Youth Serv Rev. (2018) 84:247–54. doi: 10.1016/j.childyouth.2017.11.030

[55] Okpych NJ Park S Powers J Harty JS Courtney ME . Relationships that persist and protect: the role of enduring relationships on early-adult outcomes among youth transitioning out of foster care. Soc service Rev (Chicago). (2023) 97:619–74. doi: 10.1086/724736

[56] Allen D . Contact after adoption: A longitudinal study of post-adoption contact arrangements. Br J Soc work. (2017) 47:1818–20. doi: 10.1093/bjsw/bcw035

[57] Lo AYH Grotevant HD Wrobel GM . Birth family contact from childhood to adulthood: Adjustment and adoption outcomes in adopted young adults. Int J Behav Dev. (2023) 47:283–93. doi: 10.1177/01650254231165839, PMID: 37485042 PMC10361248

[58] Grotevant HD Rueter M Von Korff L Gonzalez C . Post-adoption contact, adoption communicative openness, and satisfaction with contact as predictors of externalizing behavior in adolescence and emerging adulthood. J Child Psychol Psychiatry. (2011) 52:529–36. doi: 10.1111/j.1469-7610.2010.02330.x, PMID: 20955207 PMC3026867

[59] Maguire-Jack K Sattler K . Neighborhood poverty, family economic well-being, and child maltreatment. J Interpers Violence. (2022) 38:4814–31. doi: 10.1177/08862605221119522, PMID: 36062823 PMC10276351

[60] Rhodes JE Spencer R Keller TE Liang B Noam G . A model for the influence of mentoring relationships on youth development. J Community Psychol. (2006) 34:691–707. doi: 10.1002/jcop.20124

[61] Bovenschen I Lang K Zimmermann J Förthner J Nowacki K Roland I . Foster children’s attachment behavior and representation: Influence of children’s pre-placement experiences and foster caregiver’s sensitivity. Child Abuse Negl. (2016) 51:323–35. doi: 10.1016/j.chiabu.2015.08.016, PMID: 26412616

[62] Caron EB Weston-Lee P Haggerty D Dozier M . Community implementation outcomes of Attachment and Biobehavioral Catch-up. Child Abuse Negl. (2016) 53:128–37. doi: 10.1016/j.chiabu.2015.11.010, PMID: 26746112 PMC4939064

[63] Rubin DM Alessandrini EA Feudtner C Mandell DS Localio AR Hadley T . Placement stability and mental health costs for children in foster care. Pediatrics. (2004) 113:1336–41. doi: 10.1542/peds.113.5.1336, PMID: 15121950

[64] Tarren-Sweeney M . Retrospective and concurrent predictors of the mental health of children in care. Child Youth Serv Rev. (2008) 30:1–25. doi: 10.1016/j.childyouth.2007.05.014

[65] Liu PY Beck AF Lindau ST Holguin M Kahn RS Fleegler E . A framework for cross-sector partnerships to address childhood adversity and improve life course health. Pediatr (Evanston). (2022) 149:S1. doi: 10.1542/peds.2021-053509O, PMID: 35503315 PMC9549524

[66] McGovern R Balogun-Katung A Artis B Alderson H Brown E Diggle T . Co-producing an intervention to prevent mental health problems in children and young people in contact with child welfare services. BMC Public Health. (2024) 24:2276. doi: 10.1186/s12889-024-19770-6, PMID: 39169316 PMC11340120

[67] van den Dries L Juffer F van IJzendoorn MH Bakermans-Kranenburg MJ . Fostering security? A meta-analysis of attachment in adopted children. Child Youth Serv Rev. (2009) 31:410–21. doi: 10.1016/j.childyouth.2008.09.008

[68] Benbenishty R Davidson-Arad B López M Devaney J Spratt T Koopmans C . Decision making in child protection: An international comparative study on maltreatment substantiation, risk assessment and interventions recommendations, and the role of professionals’ child welfare attitudes. Child Abuse Negl. (2015) 49:63–75. doi: 10.1016/j.chiabu.2015.03.015, PMID: 25935254

[69] McKenna S Donnelly M Onyeka IN O’Reilly D Maguire A . Experience of child welfare services and long-term adult mental health outcomes: a scoping review. Soc Psychiatry Psychiatr Epidemiol. (2021) 56:1115–45. doi: 10.1007/s00127-021-02069-x, PMID: 33779782 PMC8225538

[70] Rutter M Sonuga-Barke EJ Beckett C Castle J Kreppner J Kumsta R . Deprivation-specific psychological patterns: effects 680 of institutional deprivation. Monogr Soc Res Child Dev. (2010) 75:i–253. Available online at: http://www.jstor.org.sire.ub.edu/stable/40608153 (Accessed August 15, 2025).

