# Supplementary material for: Developmental outcomes across foster care, adoption, and child welfare services: a mini review
Source: Front Psychiatry. 2026 Jan 29;17:1691850. doi: 10.3389/fpsyt.2026.1691850 (PMC12894384; doi:10.3389/fpsyt.2026.1691850)
Supplement: Supplementary file 1 [file Table1.docx]

**Supplementary Table S1. Summary of Core Studies (2017–2025) Included in the Narrative Synthesis**

This table summarises the 22 core studies that provided the most comprehensive and recent evidence across five developmental domains: placement stability, psychological and behavioural health, educational attainment, physical and developmental health, and social relationships and support networks. Each entry details study design, sample, care pathway, and key findings.
*Note:* Although the review covers studies published between 2020 and 2025, several longitudinal cohorts initiated before 2020 (e.g., Sonuga-Barke 2017) are included due to their continued relevance.

| **Domain** | **Study (First Author, Year)** | **Design & Sample** | **Care Pathway** | **Key Findings** |
| --- | --- | --- | --- | --- |
| **Placement Stability** | Eltink EMA et al., 2025 | Meta-analysis (47 studies, ≈ 80 000 participants) | Foster care | Mean placement breakdown ≈ 26%; highest among adolescents; behavioural issues major predictor. |
|  | Mc Grath-Lone L et al., 2020 | Longitudinal administrative data (England) | Foster care | Multiple moves associated with poorer emotional and educational outcomes. |
|  | Stenason L & Romano E, 2023 | National registry cohort (Sweden) | Foster care | Number of placement changes predicted behavioural difficulties. |
|  | Asif N et al., 2024 | Longitudinal (Pathways of Care Study, Australia) | Foster care | Placement continuity improved socio-emotional and cognitive development. |
|  | Vanderwill LA et al., 2021 | Systematic review (29 studies) | Foster & adoptive care | Carer preparation and training associated with placement permanence. |
| **Psychological & Behavioural Health** | Solerdelcoll M et al., 2022 | Retrospective controlled cohort (psychiatric inpatients) | Foster care | Elevated rates of conduct, substance-use and comorbid disorders vs controls. |
|  | Maguire D et al., 2024 | Systematic review (14 studies) | Foster care | Placement instability predicted externalising and internalising symptoms. |
|  | Mc Leigh JD et al., 2023 | Cross-sectional (US Medicaid cohort) | Foster care | High prevalence of mental-health disorders and psychotropic polypharmacy. |
|  | Yoon S et al., 2023 | Longitudinal (US adolescents) | CWS / family supervision | Secure caregiver relationships buffered behavioural risk. |
|  | Brodzinsky D et al., 2022 | Review (conceptual + empirical synthesis) | Adoption | Adoption improves wellbeing; attachment and identity issues may persist. |
|  | Sonuga-Barke EJS et al., 2017 | Longitudinal cohort (English & Romanian Adoptee Study) | Adoption (post-institutional) | Early deprivation predicted enduring neurodevelopmental difficulties. |
| **Educational Attainment** | Okpych NJ et al., 2025 | Systematic review (17 studies, 2000–2023) | Foster & care-experienced youth | 69–85 % complete secondary education; 8–12 % obtain tertiary degree. |
|  | Melkman EP, 2020 | National cohort (England) | Foster care | Early placement linked to better literacy and school attendance. |
|  | Vinnerljung B & Hjern A, 2011 | National cohort (Sweden) | Adoption vs long-term FC | Adoption predicted higher education and employment in adulthood. |
|  | Mc Namara P et al., 2019 | International review (out-of-home care) | Mixed pathways | School instability and limited support impede academic progress. |
| **Physical & Developmental Health** | Leroy JL et al., 2025 | Individual participant data meta-analysis (9 studies, n = 485) | Adoption / foster | Post-placement catch-up growth (+3 cm); ≈ 46 % developmental gap reduction. |
|  | Carrera P et al., 2025 | Neurocognitive study (adolescents, Eastern Europe) | Adoption | Persistent deficits in FASD group; improved executive function overall. |
|  | Kaferly J et al., 2024 | Administrative health data (Medicaid cohort) | Foster care | Two- to three-fold higher chronic-illness and mental-health diagnoses vs peers. |
|  | Mc Kenzie EF et al., 2025 | Linked administrative cohort (Australia) | CWS (intergenerational) | Continued health-service use across family generations under CWS. |
| **Social Relationships & Support Networks** | Okpych NJ et al., 2023 | Longitudinal (17–21 years, n ≈ 700) | Foster care | ≈ 50 % maintained enduring adult relationships; protective against homelessness. |
|  | Lo AYH et al., 2023 | Longitudinal (adopted young adults) | Adoption | Positive and sustained birth-family contact predicted better adjustment and identity integration. |
|  | Wojciak AS et al., 2018 | Mixed-methods study (US) | Foster care | Sibling connection enhanced resilience and wellbeing. |

Abbreviations: *CWS = Child Welfare Services; FASD = Foetal Alcohol Spectrum Disorder.*
